# QUALITY OF LIFE, PAIN, ANXIETY AND DEPRESSION IN PATIENTS SURGICALLY TREATED WITH CANCER OF RECTUM

**DOI:** 10.1590/S0102-67202014000200003

**Published:** 2014

**Authors:** Letácio José Freire SANTOS, João Batista dos Santos GARCIA, Jairo Sousa PACHECO, Érica Brandão de Morais VIEIRA, Alcione Miranda dos SANTOS

**Affiliations:** Study carried out in the Federal University of Maranhão, Maranhão Institute of Oncology, Hospital Aldenora Bello, and in the Oncology Unit Dr. Raymundo Matos Serrão, Hospital Dr. Tarquínio Lopes Filho, São Luis, MA, Brazil.

**Keywords:** Cancer, Pain, Life quality, Depression, Anxiety

## Abstract

**Background:**

The rectum cancer is associated with high rates of complications and morbidities with great impact on the lives of affected individuals.

**Aim:**

To evaluate quality of life, pain, anxiety and depression in patients treated for medium and lower rectum cancer, submitted to surgical intervention.

**Methods:**

A descriptive cross-sectional study. Eighty-eight records of patients with medium and lower rectum cancer, submitted to surgical intervention were selected, and enrolled. Forty-seven patients died within the study period, and the other 41 were studied. Question forms EORTC QLQ-C30 and EORTC QLQ-CR38 were used to assess quality of life. Pain evaluation was carried out using the Visual Analogical Scale, depression and anxiety were assessed through Depression Inventories and Beck's Anxiety, respectively. The correlation between pain intensity, depression and anxiety was carried out, and between these and the EORTC QLQ-C30 General Scale for Health Status and overall quality of life, as well as the EORTC QLQ-CR38 functional and symptom scales.

**Results:**

Of the 41 patients of the study, 52% presented pain, depression in 47%, and anxiety in 39%. There was a marking positive correlation between pain intensity and depression. There was a moderate negative correlation between depression and general health status, and overall quality of life as well as pain intensity with the latter. There was a statistically significant negative correlation between future depression perspective and sexual function, and also a strong positive correlation between depression and sexual impairments. A positive correlation between anxiety and gastro-intestinal problems, both statistically significant, was observed.

**Conclusion:**

Evaluation scales showed detriment on quality life evaluation, besides an elevated incidence of pain, depression, and anxiety; a correlation among these, and factors which influence on the quality of life of post-surgical medium and lower rectum cancer patients was observed.

## Abstract (pt)

**Racional:**

O câncer de reto está associado com altos índices de complicações e morbidade com grande impacto na vida dos indivíduos por ele acometidos.

**Objetivo:**

Avaliar a qualidade de vida, dor, depressão e ansiedade em pacientes com câncer de reto médio e inferior, submetidos à intervenção cirúrgica com intenção curativa.

**Métodos:**

Trata-se de estudo descritivo, transversal. Foram selecionados inicialmente 88 prontuários de pacientes com câncer de reto médio e inferior submetidos à operação radical. Destes, 47 faleceram no período e os 41 remanescentes foram convocados para o estudo. Avaliouse a qualidade de vida pelos questionários EORTC QLQ-C30 e O EORTC QLQ-CR38, e a dor através da Escala Visual Analógica; para depressão e ansiedade utilizaram-se os Inventários de Depressão e Ansiedade de Beck respectivamente. Foi feita a correlação entre intensidade da dor, depressão, ansiedade entre estes e a Escala de Estado Geral de Saúde e Qualidade de Vida Global do EORTC QLQ-C30, além das escalas funcionais e de sintomas do EORTC QLQ-CR38.

**Resultados:**

Dos 41 pacientes que compareceram, 52% apresentaram dor, 47% depressão e 39% ansiedade. Houve correlação positiva forte entre intensidade dolorosa e depressão, correlação negativa moderada entre depressão e estado geral de saúde e qualidade de vida global, e desta com a intensidade dolorosa. Houve correlação negativa estatisticamente significante entre depressão perspectiva futura e função sexual, assim como correlação positiva forte entre depressão e problemas sexuais. Observou-se correlação positiva entre ansiedade e problemas gastrointestinais e sexuais, ambos estatisticamente significantes.

**Conclusão:**

Houve prejuízo nas escalas de avaliação da qualidade de vida. Além da alta prevalência de dor, depressão e ansiedade, observou-se correlações entre estes e fatores que influenciam a qualidade de vida dos portadores de câncer de reto médio e inferior após a operação.

## INTRODUCTION

Colorectal cancer has a variable geographic distribution and is related to risk factors, such as heredity, dietary pattern, obesity and smoking^4^. It is the second most prevalent tumor worldwide^9^. In Brazil, estimate for 2012 was 30,140 new cases. From these, 14,180 would affect males and 15,960 females. In the state of Maranhão, the estimate for the same year was 200 new cases, from these 90 affecting males. It is the fourth most frequent type, being second only to prostate, lungs and stomach cancer. Among females, this is the third more frequent tumor, second only to breast and cervical cancer, with the incidence of 110 cases^23^.

The classification of rectal tumors according to their location influences neoadjuvant and/or surgical treatment. Lower third tumors (up to 5 cm of the anal margin) and medium third tumors (5.1 to 10 cm of the anal margin), if incipient, are only treated with surgery. If advanced, they are treated with radiochemotherapy plus surgical procedure. Upper third tumors (10.1 to 15 cm of the anal margin) have biological behavior and treatment similar to colon tumors^11,29^.

Better understanding of rectal cancer natural history, associated to the concept of negative lateral margin and introduction of careful dissection along embryonic planes has decreased recurrence from 37% in the 1980's to less than 10% today in specialized centers, and global survival above 60% in five years. The acceptance of 1 cm negative distal margin and the technological progress of surgical staplers have increased the number of conservative surgeries, currently around 70%-75%, preserving sphincter function and fecal continence^6,10^.

However, curative treatment of mid and low rectal cancer implies high levels of complications and morbidity, which result in long term objective and subjective changes^8^. Most important are major anatomofunctional disorders and particularities regarding behavior and patients' level of adaptation to the disease, because there is the constant threat of life expectation, in addition to conditions imposed by diagnostic procedures, symptoms and type of treatment^15,18^.

Among symptoms, pain is especially referred by cancer patients in general, and its prevalence varies with disease staging and site^14^. Specifically for rectal cancer, there is increased pain in advanced disease, with persistence in 30% of patients who require long term analgesia^15^. Due to multidimensional cancer pain aspects and complex inter-relations (physiological, psychological, cognitive and social), adequate pain evaluation and treatment are critical because pain experience of cancer patients is constantly related to distress, depression, anxiety, fear, negative mood and suicide ideas. Pain persistence increases patients' concern with regard to disease progression, especially when it is underestimated by health professionals^14,30^.

Additionally, depression and anxiety are independently present in 30%-39% of advanced cancer patients^27^. So, psychiatric disorders should be investigated because diagnosis and treatment are very often omitted in such patients, affecting their quality of life (QL), impairing functional capacity and bringing physical and emotional limitations^28^.

The dimensioning of QL and of variables influencing it in rectal cancer patients after standard therapy, also allows the evaluation and diagnosis of treatment adverse effects^8^. So, it is relevant for the daily clinical practice the adoption of approaches to minimize pain, functional damage and psychosocial repercussions, especially anxiety and depression.

In Brazil, we lack studies of this nature, which has motivated this study, the objective of which is to evaluate QL, presence of pain, anxiety and depression and their possible correlations in patients with mid and low rectal cancer submitted to surgical intervention with curative intention.

## METHODS

This study was approved by the Ethics Committee under n. 00687/10 and all patients have signed the Free and Informed Consent Term.

This is a descriptive, transversal study carried out in the Maranhão Institute of Oncology, Hospital Aldenora Bello, and in the Oncology Unit Dr. Raymundo Matos Serrão, Hospital Dr. Tarquínio Lopes Filho, both reference cancer treatment centers in São Luis, State of Maranhão, Brazil.

**Figure 1:**
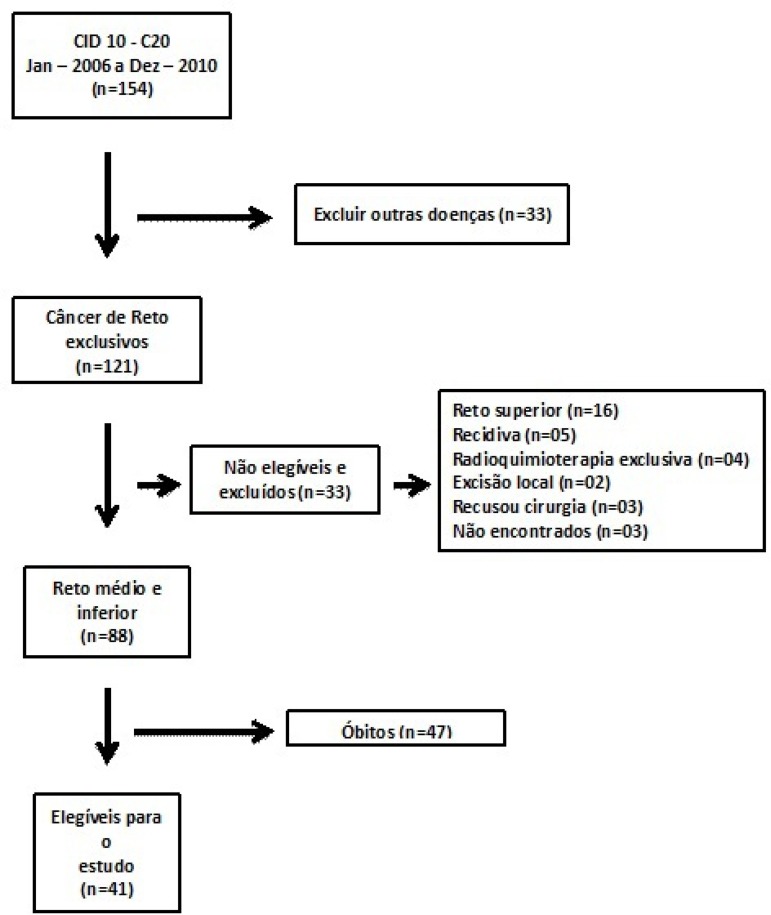
Flowchart of patients' selection process

Non probabilistic sample was made up of patients selected after a query in medical records databases of the above-mentioned hospitals, all admitted with CID-10 C20 (rectal cancer) from January 2006 to December 2010. We have selected 88 medical records of patients with mid and low rectal cancer, however 47 had already died, remaining 41 patients operated on with curative intention (anterior rectal dissection or abdominoperineal amputation with total mesorectal excision), submitted or not to neoadjuvant or adjuvant radiochemotherapy who were invited to the study (Figure 1). Exclusion criteria were patients with local or distant recurrence, those treated with local excision and those who were not found (have not returned for follow up).

As data collection sources, we have used information obtained from patients' medical records to fill the protocol card specifically developed for rectal tumors and including sociodemographic variables, data related to symptoms, diagnosis, staging, treatment and complications. After checking inclusion criteria, an outpatient clinical interview was carried out to complement the protocol card. Karnofsky status performance scale was applied^22^, in addition to validated questionnaires for QL, pain, depression and anxiety. Two questionnaires developed by the European Organization for Research and Treatment of Cancer (EORTC), validated for Brazil were used^24^.

The first, EORTC QLQ-C30, provides generic evaluation of cancer patients. It is made up of five functional scales (physical, routine, cognitive, emotional and social); three symptoms scales (fatigue, pain and nausea/vomiting); six items evaluating different aspects of global quality of life (dyspnea, insomnia, anorexia, constipation, diarrhea and financial problems); and two questions about general health status and global quality of life^1^.

The second EORTC QLQ-CR38 is an additional questionnaire evaluating specific rectal cancer symptoms and side effects related to different types of treatment. It is made up of 38 items. It incorporates two functional scales: body image and sexuality; seven symptoms scales: voiding problems, symptoms related to the gastrointestinal tract, chemotherapy side-effects, defecation problems, problems related to stoma and sexual problems. Other items are future perspective and weight loss. Both questionnaires have four alternative answers: no, mild, moderate and severe, except for the global health evaluation and quality of life scale (GHE/GQL) of the EORTC QLQ-C30 questionnaire which has seven alternatives going from lousy to excellent.

Mean results of items contributing to the scale, the Raw Score, were obtained, which suffer linear transformation in a scale from zero to 100. High functional scales scores represent high function levels, while high symptoms scales scores represent significant exacerbation of symptoms or distress^25^. To help the evaluation of such scores, it was suggested that scores above 50 would indicate satisfactory quality and function and inadequate symptoms control.

Pain intensity was measured with the Visual Analog Scale (VAS)^12^, which submits patients to a non-graded line where one end corresponds to no pain and the opposite end represents the worst imaginable pain, in addition to data about pain onset.

Beck Depression Inventory (BDI) and Beck Anxiety Inventory (BAI) were used to measure depression and anxiety, respectively. Both are adapted and validated for Brazil and have excellent reliability levels^7^. Inventories were applied according to guidelines of the Portuguese version. They are made up of 21 items, with four alternative answers, according to increasing manifestation of symptoms severity. A total score is obtained adding all items checked by the respondent. Highest possible score is 63 points. For this study, the following scores for depression were considered: absence or minimum depression (0.9), mild (10-16), moderate (17-29) and severe (30-63), while anxiety levels were: minimum (0-7), mild (8-15), moderate (16-25) and severe (26-63)^3^.

Data were organized in Excel 2007® spreadsheets and statistical tests were analyzed by the Statistical Package for Social Sciences (SPSS) version 17.0. Descriptive analysis was carried out. Absolute and relative frequency measures were used to quantify categorical variables, and central trend (mean) and variability (standard deviation) measures were used for qualitative variables. Variables distribution was checked with the Shapiro-Wilk test. When abnormalities were observed the Spearman correlation test was used. Correlation coefficient below 0.10 indicated no relation; between 0.10 and 0.30 weak relation; between 0.30 and 0.50 moderate relation and above 0.50 strong relation. Significance level for all statistical tests was 5% (p < 0.05).

## RESULTS

Questions were well accepted and understood by most patients. Sample was made especially by females. Mean age was 53.7±15.4 years. Most patients (25) lived with companion, nine were single, three divorced and four widowers. As to education, 12 were illiterate, 17 had basic education, 10 had high school and only two had completed university. From these, 23 patients were catholic and 18 evangelical.

Mean time between surgical procedure and interview was 28.7±18.7 months. Mean functional capacity evaluation (Karnofsky) was high (86.5±8.8). As to diagnosis, nine (23%) were stage I, 13 (34.2%) IIa, one (2.6%) IIb, nine (23%) IIIb and six (15.8%) IIIc. There has been predominance of medium third rectal tumors (23 or 56%). Remaining 18 cases were lower third tumors (44%). Twenty-seven patients (65.8%) were submitted to neoadjuvant radiochemotherapy and 2 (4.9%) exclusively to radiotherapy. Eight patients (19.5%) were submitted to adjuvant radiochemotherapy and 26 (63.4%) exclusively to chemotherapy. Postoperative complications were present in 14 (34%) patients. Sphincter preservation was observed in 20 (49%) patients. Pain was referred by 21 patients (52%) with mean intensity of 3.8±2.4. From these, 19 (90.5%) had pain onset three months before or longer. Twenty-two (53.7%) had minimum or absent depression, nine (21.9%) mild depression, eight (19.5%) moderate depression and two (4.9%) severe depression. Minimum anxiety level was seen in 25 (60.9%) patients, mild in 11 (26.9%) patients, moderate in 4 (9.8%) patients and severe in 1 (2.4%) patient (Table 1).

**TABLE 1:** Pain prevalence, duration and intensity, anxiety and depression classification by Beck inventory in mid and low rectal cancer patients after surgical curative treatment. São Luis, 2012.

Variables		n	%
Presence of pain			
	No	19	47.5
	Yes	21	52.5
Pain duration			
	< 3 months	2	9.5
	= 3 months	18	90.5
Pain intensity		3.8 ± 2.4*	
Depression			
	Absent or minimum	22	53.7
	Mild	9	21.9
	Moderate	8	19.5
	Severe	2	4.9
Anxiety			
	Absent or minimum	25	60.9
	Mild	11	25.9
	Moderate	4	9.8
	Severe	1	2.4

* Mean and standard deviation

Quality of life scores and EORTC QLQ-C30 scores are shown. Results of functional scales, symptoms and general health status evaluation meet satisfactory quality and function criteria and adequate symptoms control (Table 2).

**TABLE 2:** EORTC QLQ-C30 results of mid and low rectal cancer patients after curative surgical treatment. São Luis, 2012.

Variables		n	meam (Sd)
	GHS/GQL*	41	79.8 (8.1)
FUNCTIONAL			
	Physical Function	41	84.9 (22.7)
	Routine Performance	41	66.6 (30.5)
	Emotional Function	40	75.9 (21.2)
	Cognitive Function	41	87.1 (24.4)
	Social Function	41	70.5 (33.5)
SYMPTOMS			
	Fatigue	41	20.4 (24.5)
	Nausea & Vomiting	41	8.7 (20.3)
	Pain	41	22 (27.5)
SIMPLE ITEMS			
	Dyspnea	41	8.1 (19.3)
	Insomnia	41	25.6 (38.8)
	Loss of appetite	41	10.5 (22.9)
	Constipation	41	11.4 (27.5)
	Diarrhea	41	4.9 (19.1)
	Financial Problems	40	32.0 (36.7)

* General Health Status and Global Quality of Life

Results of the evaluation of functional and symptoms scales of the EORTC QLQ-CR38 questionnaire have shown more attention to sexual function and satisfaction, as well as future perspective with borderline index (Table 3).

**TABLE 3:** Description of EORTC QLQ-C38 quality of life tool for mid and low rectal cancer patients after curative surgical treatment. São Luis, 2012.

Variables		n	mean (Sd)
FUNCTIONAL			
	Body Image	40	61.4 (30.9)
	Sexual Function	40	30.4 (31.3)
	Sexual Satisfaction	41	13.0 (26.8)
	Future Perspective	41	50.9 (34.0)
SYMPTOMS			
	Voiding Problems	41	18.9 (21.0)
	Gastrointestinal Symptoms	41	4.9 (19.1)
	Chemotherapy Effects	39	11.1 (15.5)
	Sexual Problems	41	44.0 (40.7)
	Ostomy Problems	24	34.0 (18.8)
	Defecation Problems	15	28.4 (23.1)
	Weight Loss	41	16.3 (28.0)

There is strong positive correlation between pain intensity and depression, and moderate negative correlation between pain intensity and GHS/GQL. There is moderate negative statistically significant correlation between depression and GHS/GQL (Table 4).

**TABLE 4:** Correlation between pain intensity, anxiety, depression and GHS/GQL** of mid and low rectal cancer patients after curative surgical treatment. São Luis, 2012

	Pain Intensity	Depression	Anxiety	EGS/QVG**
Pain	1.0000			
Intensity				
Level of Depression	0.6100*	1.0000	0.2964	
Level of Anxiety	0.3410		1.0000	
GHS/GQL**	-0.4472*	-0.4434*	-0.2245	1.0000

* Spearman Correlation p<0.05

GHS/GQL** General Health Status and GlobalQuality of Life

In correlations between pain intensity, depression, anxiety and functional and symptoms scales of the EORTC QLQ-CR38 questionnaire, one should stress the strong negative correlation between depression and sexual function; moderate negative correlation between depression and future perspective. There is strong positive correlation between depression and sexual problems. There is moderate positive correlation between anxiety and gastrointestinal problems and moderate positive correlation between anxiety and sexual problems. All of them are statistically significant (Table 5).

**TABLE 5:** Correlation between pain intensity, depression, anxiety and functional and symptoms scales of the EORTC QLQ-CR38 questionnaire in mid and low rectal cancer patients after curative surgical treatment. São Luis, 2012.

	Pain Intensity	Depression	Anxiety
FUNCTIONAL			
Body Image	-0.0163	-0.3663	-0.1647
Sexual Function	0.0138	-0.5175*	-0.2911
Sexual Satisfaction	-0.1059	-0.3945	-0,2048
Future Perspective	-0.1347	-0.4453*	-0.2299
SYMPTOMS			
Voiding Problems	0.0410	-0.0031	-0.0183
Gastrointestinal Symptoms	-0.0683	0.2821	0.3975*
Chemotherapy Effect	0.3212	0.1583	0.2567
Sexual Problems	0, 2116	0.5198*	0.4017*
Ostomy Problems	-0.0920	-0.1999	-0.3458
Defecation Problems	0.0000	0.2043	0.3214
Weight Loss	-0.1590	0.3402	0.0526

Spearman Correlation

* p<0.05

## DISCUSSION

The prevalence of rectal cancer in the sample has shown slight predominance of females as compared to males, which is compatible with national mean^9^ and slightly different from world mean (13.1 and 7.6 for every 100 thousand inhab./year for males and females, respectively)^4^. Mean age was above 50 years and most lived with companion. In our investigation, time elapsed between surgical treatment and interview was short and variable. All participants had less than five years of follow up, which is the minimum probable time for recurrence and with major impact on QL^5^. After rectal cancer treatment, there is a trend to physical function stabilization within one year, while psychological changes predicting depression, anxiety and pain persist after this period^28^.

Our study has not investigated the association between variables characterizing the sample and QL, pain, depression and anxiety. However, one should stress that more than 70% of patients had low education (29% were illiterate), fact which negatively influences QL^26^. All patients were under outpatient follow up with preserved functionality. There has been a low number of conservative surgeries as compared to the literature^6^, and there were many patients undergoing neoadjuvant and adjuvant treatment. This is justified by the predominance of patients with advanced disease - most in stages II and III - which reflects on symptoms exacerbation and influences treatment^13^.

Functional evaluation and GHS/GQL described in the EORTC QLQ-C30 tool was good since global mean scores of functional scales were above 50 in a linear scale from zero to 100, with slight trend to lower routine performance scale. In symptoms and simple items scales values were low, with high trend to insomnia, pain and fatigue scales, with emphasis on financial problems. The opposite is described for EORTC QLQ-CR38 where impairment in function scales, especially in sexual satisfaction scale is evident. In symptoms scales, scores are higher for sexual and ostomy problems. Such data reinforce the need for the combination of specific and generic questionnaires to prevent misinterpretations or false impressions, broadening information about the effects of the disease and treatment in the long term, in spite of the limited value of a transversal study^19^.

The prevalence of depression (46.3%) was high as compared to that observed by other authors in rectal cancer patients^16,22^. There has been strong positive correlation between depression and pain intensity and moderate negative correlation between depression and GHS/GQL. There is clear relation between pain and psychosocial variables; these factors coexist making difficult the isolated evaluation of them^14^. It is known that depressive symptoms are twice more frequent when associated to pain^14^. This reciprocity relation should be taken into consideration because it negatively influences quality of life of such patients^21^. Pain prevalence was high in our sample. Most patients had symptoms for a period equal to or higher than three months and mean pain intensity was 3.8 according to VAS, maybe due to lack of specialized follow up or to placing pain complaint in a second plane due to inadequate beliefs or the fixation in curing the disease.

From 41 patients, 32 were sexually inactive in the interview period and most had sexual function deterioration after rectal cancer treatment. Correlations between depression and sexual function and problems and correlation between anxiety and sexual problems were evident. Most tumors were in advanced stage and needed neoadjuvant and/or adjuvant treatment, thus contributing to the high number of patients with colostomy and, as a consequence, bringing sexual impairment, since radiochemotherapy affects anorectal function and promotes sexual dysfunction, thus decreasing QL^17^. The influence of ostomy on QL is controversial, but there is superiority of cognitive, sexual and emotional function and vitality in those without ostomy^2^. So, strong associations of psychological problems and sexual dysfunction have been reported with high levels of depression, anxiety and other negative complaints associated to somatic manifestations^17^.

Our study has found a strong correlation between depression and future perspective, in addition to a trend to low EORTC QLQ-CR38 questionnaire scores. This is justified by the short post-treatment period of most patients. In the beginning of the therapy there is higher risk of recurrence, influenced by the severity of the disease and consequent aggressive treatment, which has been applied to most patients. During this period, there is negative influence of depression in the maintenance of interpersonal relationships, which induces patients to loneliness and hopelessness^27^. However, physcological manifestations of well-being, hope and future perspective tend to improve after 36 months of treatment^23^.

There has also been high prevalence of anxiety (39.1%), as compared to other colorectal cancer studies^20,28^, however, its correlation with pain intensity, depression and GHS/GQL was weak without statistical significance. Colorectal cancer diagnosis associated to symptoms and treatment effects promote psychological vulnerability, especially the association of depression and anxiety, inducing low QL expectation, but such results may change due to the transient characteristic of symptoms along time^28^.

The association of symptoms and psychological disorders is very evident among colorectal cancer patients. They suffer with changes in intestinal habits and other physical symptoms which impair postoperative performance. Our study has observed positive correlation between anxiety and gastrointestinal symptoms, although presenting low scores in the EORTC QLQ-CR38 questionnaire. These symptoms may be related to psychological problems persisting along the years, even after the disease is controlled^28^, that is, there is somatization or clinical manifestation of anxiety.

Our study has some limitations, such as the type of probabilistic sample, the small number of patients and the transversal retrospective design which does not evaluate cause and effect relationship, although allowing the formulation of hypotheses based on observed associations. New prospective studies should be carried out by following patients and the impact of disease and treatment along time.

## CONCLUSION

Mid and low rectal cancer patients after surgical treatment have poorer symptoms and functions scores in the evaluation of QL, which become more evident when associated to pain intensity, depression and anxiety. These data suggest the multifactorial origin of the problem, indicating the need for approaches in several fields: physiological, cognitive, emotional and social.
